# A new prognostic scale for the early prediction of ischemic stroke recovery mainly based on traditional Chinese medicine symptoms and NIHSS score: a retrospective cohort study

**DOI:** 10.1186/s12906-015-0903-1

**Published:** 2015-11-16

**Authors:** Ke-Gang Cao, Cai-Hong Fu, Huan-Qin Li, Xi-Yan Xin, Ying Gao

**Affiliations:** Department of Neurology, Dongzhimen Hospital affiliated to Beijing University of Chinese Medicine, Beijing, China; Department of Acupuncture and Moxibustion, Beijing Hospital of Traditional Chinese Medicine affiliated to Capital Medical University, Beijing, China; Traditional Chinese Medicine Department, Peking University Third Hospital, Beijing, China

## Abstract

**Background:**

Ischemic stroke (IS) is a common disease, often resulting in death or disability. Previous studies on prognosis of stroke mainly focused on the baseline condition or modern expensive tests. However, the change of clinical symptoms during acute stage is considerably neglected. In our study, we aim to develop a new prognostic scale to predict the 90-day outcome of IS patients.

**Methods:**

In this retrospective cohort study, a secondary data analysis was performed on 489 patients extracted from 1046 patients of 4 hospitals. A new prognostic scale was constructed to predict the recovery of IS mainly based on the National Institutes of Health Stroke Scale (NIHSS) score, traditional Chinese Medicine (TCM) symptoms & signs and the changes during the first 3 days of patients in the 3 TCM hospitals. Receiver Operating Characteristic (ROC) curve was used to determine the cutoff point for prediction. In the end, the scale was used to test the outcome of IS patients in Xuanwu hospital.

**Results:**

The new prognostic scale was composed of 8 items including age degree (OR = 3.32; 95 % CI: 1.72–6.42), history of diabetes mellitus (DM) (OR = 2.20; 95 % CI: 1.19–4.08), NIHSS score (OR = 3.08; 95 % CI: 2.16–4.40), anxiety (OR = 3.17; 95 % CI: 1.90–5.29) and irritability (OR = 4.61; 95 % CI: 1.36–15.63) on the 1st day of illness onset, change in NIHSS score (OR = 2.49; 95 % CI: 1.31–4.73), and circumrotating (OR = 7.80; 95 % CI: 1.98–30.64) and tinnitus (OR = 13.25; 95 % CI: 1.55–113.34) during the first 3 days of stroke onset. The total score of the scale was 16.5 and the cutoff point was 9.5, which means patients would have poor outcome at 90 days of stroke onset if the score was higher than 9.5. The new scale was validated on the data of Xuanwu hospital, and the value of its sensitivity, specificity and overall accuracy were 69.6 %, 83.3 % and 75.0 % respectively.

**Conclusions:**

The 8-item scale, mainly based on TCM symptoms, NIHSS score and their changes during the first 3 days, can predict the 90-day outcome for IS patients while it still needs to be further validated and optimized clinically.

**Electronic supplementary material:**

The online version of this article (doi:10.1186/s12906-015-0903-1) contains supplementary material, which is available to authorized users.

## Background

Ischemic stroke (IS) is a common disease with the characteristics of high incidence, high mortality, severe morbidity, high recurrence rate and serious complications, and it imposes a huge economic burden on the family and the society [1–7]. Stroke has been listed as the third leading cause of death following heart diseases and cancer in the world [3, 4, 6]. Ischemic stroke accounts for approximately 80 % of the total cerebrovascular diseases [8]. Therefore, the prevention and treatment of ischemic stroke has attracted a great attention from the medical profession domestically and internationally in recent years [9, 10]. Meanwhile the judgment of stroke prognosis plays an important role in the formulation of health and economic policy and the choice of treatment programs.

Prognosis, affecting both initial therapy and rehabilitation plans of ischemic stroke patients, has been studied for several decades [11]. Previous studies on prognosis of ischemic stroke was mainly focused on the baseline condition or some modern expensive tests like Magnetic Resonance diffusion-weighted Imaging (MR DWI) [12], as well as static signs, such as obviously unconscious [13]. Recently, some researchers have attached importance to the change of signs, such as blood pressure (BP) during the acute stage [14]. However, the change of clinical symptoms during acute stage is consistently neglected. A comprehensive and precise prognosis scale of ischemic stroke still needs to be further developed [15].

The National Institutes of Health Stroke Scale (NIHSS) is widely used to evaluate the severity of neurological deficit of stroke patients [16, 17]. Barthel Index (BI) is the most commonly used tool to evaluate the physical disability and functional recovery after stroke [18, 19]. Modern medical studies also show that these factors such as age, gender, hypertension, diabetes, smoking, and drinking alcohol, have a close relationship with the prognosis of stroke [6, 20, 21].

In Traditional Chinese Medicine (TCM), symptoms or syndromes can influence the diagnosis and treatment of the disease greatly. Many researchers analyse and induce TCM Symptoms or syndrome by means of modern algorithms to be convenient in clinical application [22–24]. Several early symptoms such as anxiety, irritability and circumrotating related to stroke outcomes were not only mentioned in ancient literature [25], but also reported by TCM doctors nowadays [26, 27]. It is important for clinical doctors to decide the treatment plan based on these symptoms & signs within the first few hours after stroke onset and predict the likely outcome of patients. Knowing the prognosis of stroke is also very important for stroke patients and their families [12, 28].

However, TCM symptoms have not been applied as the early predictive indicators of stroke outcome in the prognostic scale of stroke. Based on NIHSS score, TCM symptoms and their changes during the first 3 days of stroke onset, we developed a new scale to predict the 90-day outcome in the current study.

## Methods

### Study design

**Fig. 1:**
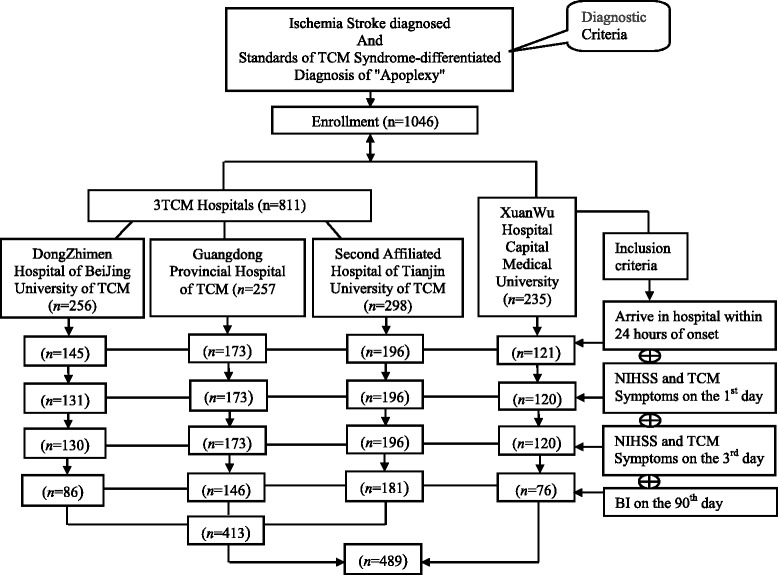
Flowchart of the study recruitment

In the retrospective cohort study, a secondary data analysis was performed on a Microsoft ACCESS database among ischemic stroke patients based on the project of Major State Basic Research Development Program of China (973 Program NO.2003CB517102) (Fig. 1).

### Ethics

The study protocol was conformed to the Helsinki Declaration [29] and the research regulations for Chinese clinical trials, which was approved by the Ethics Committee of Dongzhimen Hospital affiliated to Beijing University of Chinese Medicine.

### Study subjects

1046 cases of ischemic stroke patients were recruited between January, 2004 to December, 2009 from inpatient, outpatient, and emergency department settings in 4 hospitals including three TCM hospitals and one western medicine hospital: Dongzhimen Hospital affiliated to Beijing University of Chinese Medicine, Second Affiliated Hospital of Tianjin University of TCM, Guangdong Provincial Chinese Medicine Hospital and Xuanwu Hospital affiliated to Capital Medical University. All ischemic stroke patients received the standard treatment according to World Health Organization (WHO) criteria [30].

#### Diagnostic criteria

(1) Western Medicine diagnostic criteria of ischemic stroke referred to WHO criteria and intracranial hemorrhage was further excluded by Computed Tomography (CT) or Magnetic Resonance Imaging (MRI) scan in the hospital [30]. (2) TCM diagnostic criteria with reference to the “Standards of TCM Syndrome-differentiated Diagnosis of Apoplexy” were regulated by the Encephalopathy Accident and Emergency Collaborative Group of State Administration of Traditional Chinese Medicine in 1995 [31].

#### Inclusion criteria

(1) All patients should conform to the diagnostic criteria of ischemic stroke in Western medicine and apoplexy; (2) Ischemic stroke can be definitely diagnosed by CT or MRI scan; (3) Patients arriving in hospital within 24 h of stroke onset were included. (4) NIHSS scores, TCM symptoms & signs of the first 3 days after stroke onset and data of BI on 90th day were completely recorded.

#### Exclusion criteria

The exclusion criteria were including: (1) patients with cerebral transient ischemic attacks (TIA); (2) cerebral apoplexy caused by blood diseases, cancer or other causes; (3) accompanying some serious illnesses, such as gastrointestinal hemorrhage, heart or kidney failure, cancer, osteoarthritis and so on; (4) patients with mental disorder or severe dementia; (5) patients with obvious sequel of apoplexy.

### Data collection

The observed data including patients’ demographics (age, gender, medical history, etc.), auxiliary examination, stroke onset time and admission time, and TCM clinical symptoms & signs were recorded on the case report form (CRF) by neurologists. TCM symptoms & signs were recorded for the 1st, 2nd, 3rd day and 90th day after stroke onset. The NIHSS was performed by researchers with NIHSS certificate on the 1st, 3rd, 7th, 14th, 28th, and 90th day in the hospital, and the score were recorded. BI on the 90th day of patients was also recorded. For the accuracy and consistence of the data in collecting and testing, a monitoring system for the collection of information and standardized abstraction forms was applied to guide the data collection in case of ambiguous, conflicting, and missing information.

### Measurements

#### Outcome measurement

The BI as a reliable and valid assessment scale can be used to assess the function of ten activities in daily life [32, 33]. The maximum score of 100 indicates a full independence. Good stroke recovery was defined as BI score of 90 or higher, indicating a near full functional independence on the 90th day [34, 35].

#### Predictors

The NIHSS is widely used to measure the neurological function and predict the outcome of stroke in the clinical practice. It consists eleven graded items including level of consciousness, language function, visual fields, eye movements, facial symmetry, motor strength, sensation, coordination and so on, which has undergone extensive validation and reliable assessments [36]. Clinical nerve function defect could change dramatically during the first 48 to 72 h after stroke onset [37]. In this study, the change in NIHSS scores would be recorded from the difference of scores between the 3rd day and the 1st day of stroke onset [14].

TCM symptoms & signs with appearance rate ≥ 5 % were selected from the database in the actual data analysis. The changes in TCM symptoms & signs during the first 3 days were expressed as disappearance of symptom (disappear), no change (keep) and new appearance (appear) respectively.

In addition, the demographic and clinical factors were assessed, such as age, sex, history of stroke, hypertension, coronary heart disease (CHD), atrial fibrillation (AF), and diabetes mellitus (DM), which were defined as patients with medical treatment history.

### Statistical analysis

The data were analyzed by SPSS statistical software version 12.0 (SPSS, Chicago, USA). The measurement data such as age, NIHSS score, BI score and course of disease were expressed as mean ± standard deviation and the enumeration data such as gender and medical history were expressed as counts (percentage). Frequency comparisons were made with the method of Chi-square test. Two-group comparisons of normally distributed data were performed with the independent samples *t*-test. Mann–Whitney *U* test was used to analyze the difference of two groups for these unmatched normally distributed data. Correlation between the TCM symptoms & signs and NIHSS score was analyzed by Spearman ranked correlation coefficient. The method of logistic regression was used to further screen prognostic factors and recursive partitioning and classification tree for optimum discrimination of variables classification between good and poor recovery. The cutoff point for the prognostic model of ischemic stroke recovery was determined by Receiver Operating Characteristic (ROC) curve with maximum Youden’s index value. A probability of *P* < 0.05 was considered to be statistically significant.

## Results

### Participants

From the total 1046 subjects in the database, 489 ischemic stroke patients were finally enrolled into the study according to the diagnostic criteria and inclusive criteria, which included 413 cases from 3 TCM hospitals and 76 cases from Xuanwu hospital (Fig. 1).

### Baseline characteristics of the patients

Table 1 showed the demographics and the baseline characteristics of the 489 patients based on the outcome details with good recovery (good recovery was defined as a score of 90 or higher on the 90-day) (*n* = 278) and poor recovery (*n* = 211) measured by BI. The average age of all the patients was 65.8 years old and 63.0 % (*n* = 308) were men. The average score of NIHSS and BI of all the stroke patients were 6.2 and 77.9 respectively. There were no statistical differences (*P* > 0.05) between the 3 TCM hospitals and Xuanwu hospital in these demographics and the baseline characteristics including the history of hypertension, stroke, CHD, DM, AF, course of disease, NIHSS scores, and BI score. And the data of these hospitals were comparable.

**Table 1:** The demographic and baseline characteristics of patients

	Total			3TCM Hospital			XW Hospital		
	Overall (*n*=489)	Good (*n*=278)	Poor (*n*=211)	Overall (*n*=413)	Good (*n*=232)	Poor (*n*=181)	Overall (*n*=76)	Good (*n*=46)	Poor (*n*=30)
Age,years^a^	65.8 ±11.05	63.6 ± 10.95	68.6 ± 10.55	66.4 ± 10.80	64.0 ±10.89^b^	69.6 ± 9.82	62.0 ± 11.68	61.8 ± 11.18	62.2 ± 12.60
Male, *n* (%)	308 (63.0)	185 (66.5)	123 (58.3)	254 (61.5)	151 (65.1)	103 (56.9)	54 (71.1)	34 (73.9)	20 (66.7)
Female, *n* (%)	181 (37.0)	93 (33.5)	88 (41.7)	159 (38.5)	81 (34.9)	78 (43.1)	22 (28.9)	12 (26.1)	10 (33.3)
Hypertension,*n* (%)	304 (62.2)	174 (62.6)	130 (61.6)	251 (60.8)	143 (61.6)	108 (59.7)	53 (69.7)	31 (67.4)	22 (73.3)
Stroke History, *n* (%)	158 (32.3)	79 (28.4)	79 (37.4)	135 (32.7)	68 (29.3)	67 (37.0)	23 (30.3)	11 (23.9)	12 (40.0)
CHD,*n* (%)	115 (23.5)	62 (22.3)	53 (25.1)	101 (24.5)	51 (22.0)	50 (27.6)	14 (18.4)	11 (23.9)	3 (10.0)
DM,*n* (%)	99 (20.2)	44 (15.8)	55 (26.1)	82 (19.9)	37 (15.9)^b^	45 (24.9)	17 (22.4)	7 (15.2)	10 (33.3)
AF,*n* (%)	28 (5.7)	15 (5.4)	13 (6.2)	24 (5.8)	13 (5.6)	11 (6.1)	4 (5.3)	2 (4.3)	2 (6.7)
Onset-admission,									
hours^a^	10.3 ± 7.11	10.7 ± 7.23	9.7 ± 6.94	10.0 ± 7.17	10.2 ± 7.22	9.7 ± 7.12	11.6 ± 6.68	13.0 ± 6.90	9.38 ± 5.80
NIHSS, day 1^a^	6.2 ± 4.77	4.4 ± 3.41	8.6 ± 5.24	6.3 ± 4.60	4.5 ± 3.17^b^	8.5 ± 5.15	6.1 ± 5.67	3.9 ± 4.41	9.5 ± 5.76
BI,day90^a^	77.9 ±24.26	94.4 ± 1.60	56.0 ± 22.84	77.7 ± 24.13	94.5 ± 1.55	56.4 ± 22.59	78.3 ± 25.12	94.2 ± 1.82	53.8 ± 24.59

*TCM* traditional Chinese medicine, *XW* Xuanwu, *CHD* coronary heart disease, *DM* Diabetes Mellitus, *AF* Atrial Fibrillation, *NIHSS* National Institutes of Health Stroke Scale, *BI* Barthel Index. *Good* good recovery, *Poor* bad recovery

^a^ Data presented as mean ± SD. ^b^ There were statistical differences (*P* < 0.05) between the good and poor recovery

### The relationship between TCM symptoms and NIHSS score

In practical analysis, 57 TCM symptoms [see Additional file 1: Table S1] were selected with the appearance rate ≥ 5 % from 157 TCM symptoms & signs except tongue and pulse. Then, the relationship between these TCM symptoms & signs and NIHSS score on the 1st day of stroke onset were analysed with Pearson Correlation, and the results demonstrated that 16 of them were related to NIHSS score closely (*P* < 0.05). Moreover, the highest correlation coefficient (r^2^) was 0.451 (Table 2).

**Table 2:** The relationship between TCM symptoms & signs and NIHSS score

TCM symptom & signs	Correlation coefficient (r^2^)	*P* value
Limb flaccid paralysis	0.451	0.000
Incontinence of urinary	0.325	0.000
Cough due to drinking	0.308	0.000
Respiratory rude	0.272	0.000
Irritability	0.255	0.000
Anxiety	0.249	0.000
Analeptic	0.186	0.000
Stiffness and tension in the limbs	0.132	0.003
Purple lips	0.108	0.017
Salivation	0.090	0.047
Tinnitus	−0.099	0.028
Thirst with desire to hot drink	−0.104	0.022
Frequent micturition	−0.107	0.018
Dry mouth	−0.117	0.009
Circumrotating	−0.141	0.002
Dizziness	−0.193	0.000

*TCM* traditional Chinese medicine. Significant difference, *P* < 0.05

### Construction of the new scale

#### Screening of the prognostic factors

The new scale was developed based on the data of 3 TCM hospitals. From the general information of patients on the 1st day on admission, age, history of DM and NIHSS score were screened out as the significant prognostic indicators of ischemic stroke (Table 1).

Table 3 showed there were statistical differences (*P* < 0.05) in 14 TCM symptoms & signs on the 1st day, which were screened from 57 TCM symptoms & signs, between the good and poor recovery measured by BI from the data of 3 TCM hospitals. And the changes of NIHSS score, irritability, circumrotating, tinnitus and difficult to cough sputum during the first 3 days were also related to the outcome (Tables 3 and 4).

**Table 3:** Comparison of TCM symptoms & signs between good and poor recovery for 3 TCM hospitals

Factors	Good (*n* = 232)	Poor (*n* = 181)	*P* value^*^
Spiritlessness, day 1,*n* (%)	107 (46.1)	105 (58.0)	0.016
Anxiety day 1,*n* (%)	49 (21.1)	94 (51.9)	0.000
Cough due to drinking, day 1,*n* (%)	48 (20.7)	69 (38.1)	0.000
Anorexia,day 1,*n* (%)	27 (11.6)	39 (21.5)	0.006
Salivation, day 1,*n* (%)	22 (9.5)	31 (17.1)	0.021
Analeptic, day 1,*n* (%)	17 (7.3)	27 (14.9)	0.013
Limb flaccid paralysis, day 1,*n* (%)	11 (4.7)	28 (15.5)	0.000
Thirst with desire to hot drink, day 1,*n* (%)	25 (10.8)	9 (5.0)	0.033
Circumrotating, day 1,*n* (%)	25 (10.8)	8 (4.4)	0.018
Emaciation,*n* (%)	9 (3.9)	24 (13.3)	0.000
Stiffness and tension in the limbs, day 1,*n* (%)	12 (5.2)	22 (12.2)	0.010
Tinnitus, day 1,*n* (%)	23 (9.9)	5 (2.8)	0.004
Irritability, day 1,*n* (%)	5 (2.2)	18 (9.9)	0.001
Incontinence of urinary, day 1,*n* (%)	10 (4.3)	19 (10.5)	0.015

Significant difference, *P* < 0.05

Good: good recovery; Poor: bad recovery

^*^ *P* values based on Chi-square test

**Table 4:** The changes of NIHSS and TCM symptoms between good and poor recovery for 3 TCM hospitals

Factors	Total (*n* =413) Change status	Good (*n* = 232)	Poor (*n* = 181)	*P* value
NIHSS	-	0.54 ± 1.80	−0.45 ± 2.74	0.000^a^
TCM symptoms & signs				
Irritability	appear,*n* (%)	0 (0.0)	3 (1.7)	0.037
	keep,*n* (%)	230 (99. 1)	166 (91.7)	
	disappear,*n* (%)	2 (0.9)	12 (6.6)	
Circumrotating	appear,*n* (%)	0 (0.0)	4 (2.2)	0.007
	keep,*n* (%)	220 (94.8)	174 (96.1)	
	disappear,*n* (%)	12 (5.2)	3 (1.7)	
Tinnitus	appear,*n* (%)	1 (0.4)	2 (1.1)	0.002
	keep,*n* (%)	219 (94.4)	179 (98.9)	
	disappear,*n* (%)	12 (5.2)	0 (0.0)	
Difficult to cough sputum	appear,*n* (%)	4 (1.7)	7 (3.9)	0.031
	keep,*n* (%)	221 (95.3)	173 (95.6)	
	disappear,*n* (%)	7 (3.0)	1 (0.6)	

*TCM* traditional Chinese medicine. *NIHSS* National Institutes of Health Stroke Scale

^a^Mann–Whitney *U* test; Good: good recovery; Poor: bad recovery

**Fig. 2:**
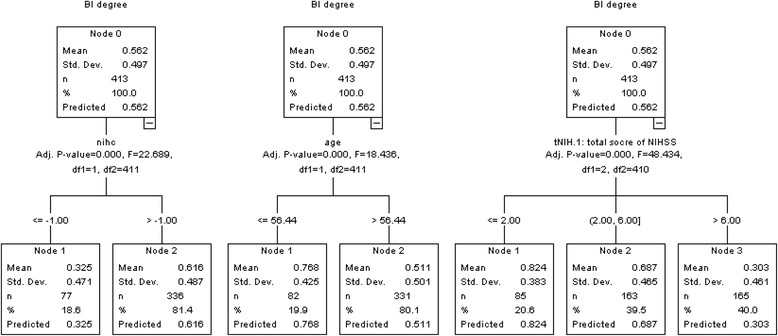
The optimum discrimination of some independent prognosis variables by the method of Classification Tree

Among the selecting factors above, the age, NIHSS score on the 1st day and the change of NIHSS score during the first 3 days were dealt with as continuous variables for optimum discrimination between good and poor recovery by the method of classification tree [12]. The analysis results showed that the two categories of age degree were assigned ≤ 56.44 or > 56.44, the three categories of NIHSS score (on the 1st day) ≤ 2, 3–6, or > 7, the two categories of change in NIHSS score (during the first 3 days) ≤ −1, or > −1 (Fig. 2).

The method of logistic regression was used to further screen prognostic factors from all variables in Tables 1, 3 and 4. On statistical analysis, there were 8 independent prognosis variables were included in the prognosis model: age [Odds ratio (OR) 3.32, *P* < 0.05], history of DM (OR 2.20, *P* < 0.05), anxiety (OR 3.17, *P* < 0.05), NIHSS score (OR 3.08, *P* < 0.05), irritability on the 1st day (OR 4.61, *P* < 0.05), and the changes in NIHSS score (OR 2.49, *P* < 0.05), circumrotating (OR 7.80, *P* < 0.05) and tinnitus (OR 13.25, *P* < 0.05) during the first 3 days (Table 5).

**Table 5:** Screening prognostic fators by the method of logistical regression model in 3 TCM hospitals

Factor	Coefficient (S.E.)	OR (95 % CI)	*P* value
Age degree	1.20 (0.34)	3.32 (1.72–6.42)	0.000
History of DM	0.79 (0.32)	2.20 (1.19–4.08)	0.012
Anxiety, day 1	1.15 (0.26)	3.17 (1.90–5.29)	0.000
NIHSS score degree, day 1	1.12 (0.18)	3.08 (2.16–4.40)	0.000
Irritability, day 1	1.53 (0.62)	4.61 (1.36–15.63)	0.014
Change in NIHSS score during 3 days	0.91 (0.33)	2.49 (1.31–4.73)	0.006
Change in circumrotating during 3 days	2.05 (0.70)	7.80 (1.98–30.64)	0.003
Change in tinnitus during 3 days	2.58 (1.10)	13.25 (1.55–113.34)	0.018

*NIHSS* National Institutes of Health Stroke Scale, *SE* standard error, *CI* confidence interval, *OR* odds ratio

#### Adding weight value for each prognostic factors

In order to use the prognostic model conveniently and effectively in clinic, different points were assigned for the categories of the 8 factors by a simple weighting scheme according to the value of findings and analysis. The results of categories obtained were as follows: age degree [0 (≤56.44); 1 (>56.44)], history of DM [0 (no); 1 (yes)], anxiety on the 1st day [0 (no); 1 (yes)], NIHSS score degree on the 1st day [0 (≤2); 1 (3–6); 2 (>7)], irritability on the 1st day [0 (no); 1.5 (yes)], change in NIHSS score during the first 3 days [0 (≤ − 1), 1 (> − 1)], change in circumrotating during the first 3 days [0 (disappear); 2 (keep); 4 (appear)], change in tinnitus during the first 3 days [0 (disappear); 2.5 (keep); 5 (appear)]. The total score was calculated by adding up all factor scores. The maximum score was 16.5, and the range of score was from 0 to 16.5 points (Table 6).

**Table 6:** A 8-item scale for the prediction of stroke recovery

Item	Weight	Assigned points
Age	1	0 (≤56.44); 1 (>56.44)
History of DM	1	0 (no); 1 (yes)
Anxiety, day 1	1	0 (no); 1 (yes)
NIHSS score degree, day 1	1	0 (≤2),1 (3–6), 2 (>7)
Irritability,day 1	1.5	0 (no); 1.5 (yes)
Change in NIHSS score during 3 days	1	0 (≤ − 1),1 (> − 1)
Change in circumrotating during 3 days	2	0 (disappear); 2 (keep); 4 (appear)
Change in tinnitus during 3 days	2.5	0 (disappear); 2.5 (keep); 5 (appear)
Total score (sum of assigned points)	-	0–16.5

*NIHSS* National Institutes of Health Stroke Scale

Then a new 8-item scale for the early prediction of ischemic stroke recovery was developed to be used for clinical assessment. It means that the older patients, with a history of DM, high NIHSS score, anxiety, irritability on the 1st day, and with an increased NIHSS score, appearance of circumrotating and tinnitus during the first 3 days, were inclined to get poor recovery.

#### Selection of cutoff value

**Fig. 3:**
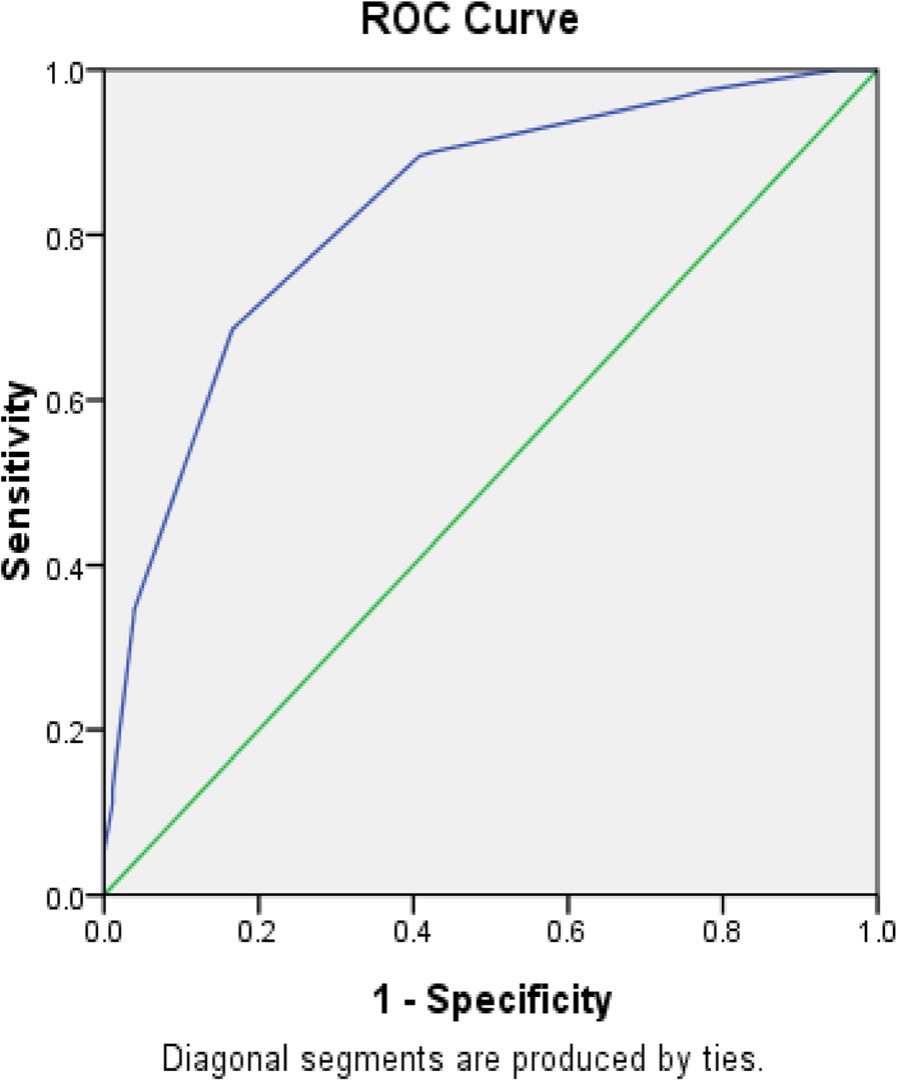
The cutoff value for predicted probability of the stroke prognosis

Receiver operating characteristic (ROC) curve analysis was performed to determine the cutoff value for the prognostic model of ischemic stroke recovery by using these 8 independent factors. The new scale was applied to estimate the prognosis of 413 ischemic stroke patients in 3 TCM hospitals by ROC curve analysis. And with prognostic assessment of ischemic stroke recovery measured by BI as the standard, the ROC curve indicated that at the cutoff point of 9.5 score, it had the greatest prognostic ability (maximum sensitivity and specificity) (Fig. 3).

The results of 310 cases (good =159, poor = 151) in the 3 TCM hospitals were predicted correctly, while the other 103 cases (good =30, poor = 73) were wrong classified. The sensitivity of this model for prediction of good recovery was 68.5 % and the specificity was 83.4 %. The overall probability of accurate prediction was 75.1 %. Therefore, the score of 9.5 was chosen as the cutoff value for prediction good and poor stroke recovery (Table 7).

**Table 7:** An 8-item scale for the prediction of stroke recovery in TCM hospitals and Xuanwu hospital

Hospital	Good, *n*	Poor, *n*	Sensitivity (%)	Specificity (%)	Accuracy (%)
	TG	FP			
	FG	TP			
3TCM Hospitals	159	73	68.5	83.4	75.1
	30	151			
XW Hospital	32	14	69.6	83.3	75.0
	5	25			

*TG* ture good recovery, *FG* false good recovery, *TP* ture poor recovery, *FP* false poor recovery

Sensitivity = TG/ (TG + FP); Specificity = TP/ (TP + FG); Accuracy = (TG + TP)/ (TG + FG + TP + FP)

### Validation of the scale for patients in Xuanwu hospital

The validation result of the new scale showed that 57 cases (good = 32, poor = 25) were predicted correctly, while the other 19 cases (good = 5, poor = 14) were wrong classified in Xuanwu hospital. The sensitivity and specificity of the scale for prediction of good recovery were 69.6 and 83.3 %, and the overall percentage of correct prediction was 75.0 % (Table 7).

## Discussion

### Summary of main findings

The study showed that NIHSS score had a relationship with some TCM symptoms & signs (*P* < 0.05), such as limb flaccid paralysis, anxiety, dizziness and so on. NIHSS is one of the most common assessment methods to predict the outcome of ischemic stroke. It found that there was some relationship between NIHSS and certain TCM symptoms which indicates TCM symptoms may have a predictive value for the outcome of ischemic stroke. Many studies have shown that age is an independent factor in predicting patients’ outcome [38, 39]. Consistent with previous researches, the result showed that patients aged 56.44 years old or younger were more likely to recover than those over 56.44 years old (OR =3.32, 95 % CI 1.72–6.42). Several studies have reported that NIHSS score had a significant effect on ischemic stroke outcome [16, 17]. The study showed that the scale could make a more accurate prognosis using the change in NIHSS scores. The patients with an increased NIHSS score more than 1 during the first 3 days were 2.49 times (OR = 2.49, 95 % CI 1.31–4.73) more likely to get poor recovery. Some TCM symptoms and signs play an important role in prognosis. For example, the patients with anxiety on the 1st day were more likely to get poor recovery than those without it (OR =3.17, 95 % CI 1.90–5.29). The disappearance of some TCM symptoms & signs such as circumrotating and tinnitus during the first 3 days can also indicate good recovery.

Finally the new scale was composed of age degree, history of DM, NIHSS score, anxiety and irritability on the 1st day, and change in NIHSS score, circumrotating and tinnitus during the first 3 days (Table 8). The total score of the scale was 16.5 and the cutoff point was 9.5 by the ROC curve, which means patients would have poor recovery outcome on the 90th day if the score >9.5. The new scale was validated on Xuanwu hospital patients, and the sensitivity of the scale for the prediction of good recovery was 69.6 % and the specificity was 83.3 % with an overall accuracy 75.0 %.

**Table 8:** The new prognosis scale for the prediction of ischemic stroke recovery

Item	Weight	Classify	Assigned point
Age degree	1.0		
≤56.44		0	0
>56.44		1	1.0
History of Diabetes	1.0		
No		0	0
Yes		1	1.0
Anxiety, day 1	1.0		
No		0	0
Yes		1	1.0
NIHSS score, day 1	1.0		
≤2		0	0
2 < NIHSS score ≤ 6		1	1.0
>6		2	2.0
Irritability, day 1	1.5		
No		0	0
Yes		1	1.5
NIHSS score change during 3 days	1.0		
≤ − 1		0	0
> − 1		1	1.0
Change in circumrotating during 3 days	1.0		
Disappear		0	0
Keep		1	2.0
Appear		2	4.0
Change in tinnitus during 3 days	2.5		
Disappear		0	0
Keep		1	2.5
Appear		2	5.0
Total score (sum of assigned points)^a^			16.5

^a^The range of score from 0 to 16.5 points; the cutoff point was 9.5

### Strengths of the study

#### To construct the scale by the TCM symptoms and NHISS

The four diagnostic methods in TCM include observation, listening and smelling, inquiry and pulse feeling diagnosis. These methods are not only the principal methods for collecting case information comprehensively, but also an important evidence for TCM differential syndrome and treatment, as well as a solid foundation for TCM clinical research and TCM clinical application [40]. Upholding TCM holism concepts as our guide, it can be of great significance to test the prognosis and recurrence of disease and guide the clinical practice by use of the comprehensive information. NIHSS scores can be used to measure the severity of stroke, which is highly predictive to excellent or devastating outcomes in ischemic stroke patients [17, 41]. It also emphatically reflects the disease outcome at objective injury level, which has been considered a close association with prognosis [42]. The study combined the TCM diagnostic information with the evaluation tool of dysfunction in modern medicine to develop the scale, so as to be both comprehensive and focused. The 8-item scale for stroke outcome can comprehensively evaluate the prognosis from physical and psychological aspects without high cost. The study would provide a relatively comprehensive evaluation tool for clinical practice.

In addition, one previous study has shown that the prognostic model based on age and NIHSS score has correctly predicted survival and functional recovery after 3 months, which provided the basis for our research [11]. The modified Rankin Scale (mRS) is the most comprehensive and most widely used scale for measuring clinical outcome of stroke patients. However, substantial inter observer variability in mRS scoring has been reported in a recent study, and the Rankin Focused Assessment (RFA) is suggested to be used in clinical trials as a short and practicable structured assessment tool [43]. Barthel index (BI) is a kind of activities of daily living (ADL) assessment tool with high reliability and sensitivity, commonly used in rehabilitation institutions of the United States. So BI was chosen as the prognostic assessment index in this study.

#### The scale reflects the dynamic change in prognosis of ischemic stroke

Stroke is a kind of disease with the characteristics of obvious stage change of symptoms and syndrome (acute phase/recovery/sequel period), which is manifested by acute onset and quick change [42]. The wind, fire, phlegm, blood stasis, and deficiency are the main syndrome factors of stroke. While NIHSS is mainly used for evaluating the degree of nerve function defect of acute ischemic stroke patients [17, 41], TCM symptoms and NIHSS reflect the development of stroke from different angles. Therefore, correlation researches on dynamic evolution of stroke syndrome elements and neurological function deficit have been conducted [44, 45]. Some researchers believe that there are certain and complex nonlinear relations between the two, while others have found they have correlations among them by use of the analysis method of Bayesian network, and the related degree would dynamic change over time [42]. Moreover, the result of the study was identical with the above conclusions. As a result, the new scale conforms to the characteristics of the dynamic development of the disease, and would more accurately evaluate the prognosis of stroke.

### Limitation of the study

The study has three limitations. Firstly, the patients are relatively milder in ordinary ward because the patients died or with incomplete record without BI data on the 90th day are not included in our study. Secondly, based on the second data analysis, the new scale needs to be further tested by prospective and lager sample size studies. Thirdly, further sensitivity tests and specificity by expert questionnaire and clinical verification is required.

## Conclusions

The 8-item scale can be used to predict the 90-day outcome for ischemic stroke patients shortly after admission to hospital, but further clinical verification researches should be conducted to make the prediction rule more dependable for clinical use.

## Additional file

Additional file 1: Table S1. TCM symptoms & signs with appearance rate no less than 5 %. In practical analysis we selected 57 TCM symptoms with the appearance rate ≥5 % from 157 TCM symptoms& signs except tongue and pulse. (CSV 1 kb)
